# Multicenter international experience with the Hugo™ RAS platform in colorectal surgery: reproducibility and real-world outcomes from the COLOROBOT IBERICA study

**DOI:** 10.1007/s11701-026-03243-6

**Published:** 2026-03-05

**Authors:** Luis Sánchez-Guillén, Pedro Brandao, Juan Manuel Romero-Marcos, Janine Tabet-Almeida, Carlos Javier Gómez-Díaz, Gloria Báguena-Requena, Marisa Santos, Salvadora Delgado-Rivilla, Manuel López-Bañeres, Pablo Collera-Ormazabal, Francisco Javier Blanco-González, Xavier Barber, Antonio Arroyo

**Affiliations:** 1 Colorectal Surgery Unit, General University Hospital of Elche, Elche, Spain; 2 Colorectal Unit, Centro Hospitalar Universitario de Santo Antonio, Porto, Portugal; 3 Department of General Surgery, Hospital Universitari MútuaTerrassa, Plaça del Doctor Robert, 5, Terrassa, Barcelona, 08221 Spain; 4 Colorectal Unit, Hospital Arnau de Vilanova de Valencia, Valencia, Spain; 5 Colorectal Unit, ALTHAIA- Xarxa Assistencial Universitària de Manresa - Sant Joan de Déu Hospital, Manresa, Spain; 6 Colorectal Unit, Hospital Universitario La Ribera de Alzira, Alzira, Spain; 7 Pathology and Surgery Department., Miguel Hernández University, Elche, Spain; 8 Center of Operations Research, Miguel Hernández University, Elche, Spain; 9 Joint Research Unit , UMH-FISABIO (STATSALUT), Elche, Spain

**Keywords:** Robotic-assisted surgery, Hugo™ RAS, Colorectal surgery, Multicenter study, Anastomotic leak

## Abstract

Robotic-assisted surgery has become increasingly relevant in colorectal procedures, particularly with the emergence of new modular platforms. Multicenter real-world data on the Hugo™ Robotic-Assisted Surgery (RAS) system remains limited. The COLOROBOT IBERICA study is a multicenter retrospective analysis including 285 consecutive patients who underwent elective robotic colorectal surgery using the Hugo™ RAS system between April 2023 and December 2024 across six centers in Spain and Portugal. Demographic data, intraoperative variables, and postoperative outcomes were analyzed. Complications were graded according to the Clavien–Dindo classification, and multivariable analyses were performed to identify predictors of operative time and adverse outcomes. A total of 285 patients were included (median age 69 years; 53% male). Median total operative time was 210 min (IQR 160–243), with a median docking time of 6 min (IQR 5–9) and a median console time of 121 min (IQR 95–146). Estimated blood loss was ≤ 500 mL in 92% of cases. Conversion rate was 1.1%. Overall postoperative complications occurred in 19.2% of patients, with major complications (Clavien–Dindo ≥ III) in 8.0%. Anastomotic leakage occurred in 5.6%, and 4.5% of patients required reintervention. Anticoagulation and neoadjuvant therapy were independently associated with increased morbidity and anastomotic leakage. No robot-related setup factors were associated with complication severity. In this large multicenter real-world cohort, the Hugo™ RAS platform enabled safe, effective, and reproducible colorectal surgery with low conversion rates and acceptable morbidity across different institutions and surgeon profiles. These findings support the scalable integration of modular robotic technology into routine colorectal practice.

## Introduction

Minimally invasive surgery has become the standard of care in colorectal procedures, offering well-established benefits such as reduced postoperative pain, shorter hospital stay, and faster recovery. Over the past two decades, robotic-assisted surgery (RAS) has emerged as a valuable alternative to conventional laparoscopy, providing enhanced dexterity, tremor filtration, superior ergonomics, and high-definition three-dimensional vision—advantages that are particularly relevant for complex pelvic dissections and low rectal procedures [1].

The da Vinci^®^ system has dominated the robotic landscape for years, but new multi-port, modular platforms have been developed to improve flexibility, ergonomics, and cost-efficiency. Among these, the Hugo™ Robotic-Assisted Surgery (RAS) system (Medtronic) features independent arm carts and an open, heads-up console that allow individualized trocar placement, improved communication, and easier teaching [2]. Since its first clinical use in 2021, the system has gained regulatory approvals worldwide, with encouraging results reported in gynecologic, urologic, and general surgery for several procedures [3–25]. Recent systematic and scoping reviews have confirmed its feasibility, reproducibility, and safety across specialties, particularly in general and colorectal surgery [26].

Despite these promising early experiences, robust multicenter data on the Hugo™ RAS platform remain scarce. The COLOROBOT IBERICA study was designed to address this evidence gap by evaluating real-world outcomes of elective colorectal procedures performed with Hugo™ across multiple centers in Spain and Portugal. This study aimed to provide a comprehensive and externally validated assessment of the system’s safety, performance, and reproducibility in diverse clinical settings, contributing to evidence-based integration of modular robotic technology into modern colorectal practice.

## Methods

### Population

Data from patients who underwent elective abdominal colorectal robotic surgery with the Hugo™ RAS system were collected across multiple colorectal units in Spain and Portugal, as part of the COLOROBOT IBERICA study. The procedures were performed between the start of each center’s robotic program and December 2024, within the framework of routine clinical practice.

Patient selection for robotic colorectal resection with the Hugo™ RAS system depended on surgeon availability, access to trained surgical teams, and platform scheduling, given that Hugo™ is also used in many centers for other robotic procedures (e.g., urological or gynecological surgeries). All included procedures were scheduled electively and performed by teams who had received prior training in the use of the Hugo™ robotic system, although many did not have specific experience in robotic colorectal surgery. Included procedures comprised a range of colorectal resections such as right and left colectomies, sigmoidectomies, low anterior resections, and abdominoperineal resections, all performed via a robotic approach with the Hugo™ system. The indication for surgery was primarily colorectal cancer, although some cases included benign pathology such as diverticular disease or endometriosis. Perioperative variables, complication definitions, and oncologic descriptors followed contemporary usage in the RAS literature to facilitate comparability with prior reports and reviews of multi-port platforms and Hugo™ technical characteristics [1, 2]. Port placement, docking geometry, and arm-cart configurations were standardized per institutional protocols drawing on published descriptions of Hugo-specific docking angles and multi-docking strategies for pelvic and multiquadrant colorectal surgery [17, 22]. Training and adoption at participating centers followed structured pathways previously reported for non-robotic teams transitioning to Hugo™ colorectal procedures, including simulation and proctored cases [16].

This multicenter study was conducted in compliance with the principles of Good Clinical Practice and the Declaration of Helsinki. All patients signed an informed consent prior to surgery, and the study protocol was approved by the ethics committees of each participating institution according to national regulations.

### Surgeon experience

Procedures included in this study were performed by 16 colorectal surgeons across six tertiary centers. Surgeon experience was formally assessed through a structured survey completed by all participating surgeons. Surgeon experience in laparoscopic colorectal surgery and robotic surgery before implementation of the Hugo™ RAS platform was categorized according to prior exposure to any robotic system. The study cohort intentionally included procedures performed from the initiation of each center’s Hugo™ RAS program, thereby encompassing both early learning-curve cases and later, more consolidated experience.

### Endpoints and variables

The primary endpoint of the study was to assess the surgical and clinical outcomes of the Hugo™ RAS system in elective colorectal resections across multiple centers and surgeons with varying levels of experience, by evaluating intraoperative and postoperative variables. The secondary endpoint was to identify risk factors associated with adverse outcomes, such as prolonged operative time or hospital stay, and increased postoperative morbidity.

Preoperative data included patient demographics (age, sex, BMI), comorbidities (hypertension, diabetes, dyslipidemia), and ASA classification. Intraoperative variables and times were recorded as draping time (from anesthesia to sterile field ready), docking time (from sterile field ready to instrument insertion), console time (from first to last robotic control), and total operative time or skin-to-skin time, estimated blood loss and conversion to laparoscopic or open surgery. Postoperative data encompassed length of hospital stay, reinterventions, readmissions, anastomotic leakage and mortality.

Postoperative complications were classified according to the Clavien-Dindo system. Anastomotic leakage (AL) was defined as a clinically suspected and/or radiologically confirmed defect of the intestinal wall integrity at the anastomotic site, leading to a change in clinical management. Postoperative imaging (computed tomography) was performed on a symptom-driven basis and not routinely. AL was classified as early when diagnosed within 30 postoperative days. Categorical variables were summarized as frequencies and percentages, and continuous variables (e.g., age, BMI, operative time) were reported as medians and interquartile ranges (IQRs).

Continuous data are presented as mean (SD) when normally distributed, or as median (IQR) otherwise. R software (Version 4.1.3, R Foundation for Statistical Computing, Vienna, Austria) was used for the statistical analysis. Categorical variables were compared using the Pearson test (X^2^) or the Fisher exact test, depending on the expected frequencies. Distribution of continuous variables was assessed using the Kolmogorv-Smirnov test. Continuous variables were compared using the Student test (t) for those which had a normal distribution or the Mann-Whitney test (U) for the rest. Statistical significance was set at *p* < 0.05. Variables showing a p value under 0.1 were included in a multivariate binary logistic regression analysis.

## Results

From April 2023 to December 2024 a total of 285 patients underwent elective abdominal colorectal robotic surgery using the Hugo™ RAS system across 6 centers in Spain and Portugal, performed by 16 different surgeons. Fifteen surgeons (93.7%) had extensive laparoscopic colorectal experience (> 100 procedures), and one had moderate experience (70–100 procedures), all routinely performed rectal surgery, and none had prior robotic experience before Hugo™ RAS adoption.

### Demographic and preoperative characteristics

The median age was 69 years (IQR 59–78), with 154 patients (54%) being male. The median BMI was 26.160 kg/m² (IQR 5.8). Hypertension was present in 147 patients (51.6%), diabetes mellitus in 62 patients (21.8%), and dyslipidemia in 119 patients (41.8%). Most patients were classified as ASA II (172; 60.4%) and ASA III (86; 30.2%). Oncological characteristics are described in Table 1.

**Table 1:** Surgical and pathological details for the whole group of patients

Variable		*N* (%)
Sex	Female	131 (46%)
	Male	154 (54%)
BMI	Median	26.16 (23.17–29.00)
Arterial hypertension	No	138 (48.8%)
	Yes	147 (51.6%)
Dyslipidemia	No	166 (58.2%)
	Yes	119 (41.8%)
Diabetes Mellitus	No	223 (78.2%)
	Yes	62 (21.8%)
Smoker	No	236 (82.8%)
	Yes	49 (17.2%)
Anticoagulation	No	249 (87.4%)
	Yes	36 (12.6%)
Chronic kidney disease	No	271 (95.1%)
	Yes	14 (4.9%)
Liver disease	No	276 (96.8%)
	Yes	9 (3.2%)
Chronic obstructive pulmonary disease	No	252 (88.4%)
	Yes	33 (11.6%)
Cardiovascular patient	No	242 (84.9%)
	Yes	43 (15.1%)
Corticosteroids intake	No	279 (97.9%)
	Yes	6 (2.1%)
Other concomitant neoplasms	No	273 (95.8%)
	Yes	12 (4.2%)
Previous abdominal surgery	No	199 (69.8%)
	Yes	86 (30.2%)
ASA level	I	22 (7.7%)
	II	172 (60.4%)
	III	86 (30.2%)
	IV	5 (1.8%)
Charlson Comorbidity Index	Median	5 (3.00–6.00)
Diagnosis	Ulcerative Colitis	1 (0.35%)
	Crohn’s disease	4 (1.4%)
	Diverticulosis	16 (5.6%)
	Rectal prolapse	18 (6.3%)
	Colon cancer	201 (70.5%)
	Rectal cancer	45 (15.7%)
Neoadjuvant therapy	No	263 (92.3%)
	Yes	22 (7.7%)
Type of surgery	Abdominoperineal amputation	4 (1.4%)
	Subtotal colectomy	1 (0.4%)
	Total colectomy +/- reservoir	3 (1.1%)
	Right hemicolectomy	104 (36.5%)
	Extended right hemicolectomy	7 (2.5%)
	Left hemicolectomy	10 (3.6%)
	Ventral rectopexy	16 (5.6%)
	Anterior resection of the rectum	66 (23.2%)
	Ileocecal resection	5 (1.8%)
	Segmental resection	2 (0.7%)
	Sigmoidectomy	67 (23.5%)
T	Tis	114 (40%)
	T1	24 (8.42%)
	T2	46 (16.1%)
	T3	92 (32.2%)
	T4a	8 (2.8%)
	T4b	1 (0.4%)
N	N0	219 (76.8%)
	N1a	23 (8.1%)
	N1b	22 (7.7%)
	N1c	4 (1.4%)
	N2a	11 (3.9%)
	N2b	3 (1.1%)
	Nx	3 (1.1%)
M	M0	247 (86.6%)
	M1	4 (1.4%)
	Mx	34 (11.9%)

BMI, body mass index; ASA, American Society of Anesthesiologists classification; T, tumor stage; N, nodal stage; M, metastasis stage

### Surgical approach and technical characteristics

All procedures were colorectal surgeries performed using the Hugo™ modular robotic platform. The median total operative time was 210 min (IQR 160–243), with a median docking time of 6 min (IQR 5–9) and a median console time of 121 min (IQR 94.75–146). Estimated intraoperative blood loss was ≤ 500 mL in 262 patients (92%). The conversion rate was 1.1%, 2 procedures to laparoscopic surgery (due to robotic trocars badly positioned) and 1 procedure to open surgery (due to intraoperative complications). Intraoperative complications were reported in 8 patients (2.9%), with the most frequent being bleeding, although most events were managed without conversion (Tables 2 and 3).

**Table 2:** Linear regression results docking, console and total surgery time

**Characteristic**		Distribution of variables	Linear regression		
		*N* = 285^1^	**Beta**	**95% CI**	**p-value**
*Linear regression results for docking time*					
Number of Trocars Used	3–4	60 (21%)	–	–	
	5–6	225 (79%)	1.5	0.10, 2.9	0.037
Height		165 (159–171)	0.23	− 0.01, 0.47	0.059
BMI		26.7 (23.17–29.00)	0.66	− 0.02, 1.3	0.058
Chronic obstructive pulmonary disease	No	252 (88.4%)	–	–	
	Yes	33 (11.6%)	− 1.1	− 2.6, 0.30	0.12
*Linear regression results for console time*					
Clavien Dindo	<=II	262 (91.9%)	–	–	
	> II	23 (8.1%)	20	− 3.4, 43	0.093
Total number of lymphadenopathies		17 (12–26)	1.3	0.36, 2.2	0.007
Neoadjuvant Therapy	No	263 (92.3%)	–	–	
	Yes	22 (7.7%)	32	4.7, 59	0.022
*Linear regression results for total surgery time*					
Charlson’s Comorbidity Index		5.00 (3.00–6.00)	5.3	− 2.3, 13	0.2
Dyslipidemia	No	166 (58.2%)	–	–	
	Yes	119 (41.8%)	− 27	− 59, 6.2	0.11
Chronic obstructive pulmonary disease	No	252 (88.4%)	–	–	
	Yes	33 (11.6%)	− 37	− 76, 2.5	0.066
Neoadjuvant Therapy	No	263 (92.3%)	–	–	
	Yes	22 (7.7%)	39	− 7.7, 85	0.10

^1^n (%); Median (IQR)

CI = Confidence Interval. BMI, body mass index; HTA, hypertension; DL, dyslipidemia; DM, diabetes mellitus; COPD, chronic obstructive pulmonary disease

**Table 3:** Morbidity details for the whole group of patients

		*N* (%)
*Intraoperative Complications*		
No complications		277 (97.1%)
Complications	Bleeding	3 (1%)
	Tumour complication	1 (0.4%)
	Bowel injuries	1 (0.4%)
	Others	3 (1%)
P*ostoperative Complications*		
No complications		212 (74.4%)
Surgical	Stoma complications	1 (0.4%)
	Evisceration	1 (0.4%)
	Paralytic ileus	6 (2.1%)
	Surgical site infection	1 (0.4%)
	Anastomotic leak	16 (5.5%)
Medical	Catheter-related bacteraemia	2 (0.7%)
	ARF	4 (1.4%)
	UTI	8 (2.8%)
	Pneumonia	4 (1.4%)
	Other medical complications	26 (9.1%)
Clavien-Dindo	I	17 (5.9%)
	II	15 (5.2%)
	IIIa	9 (3.2%)
	IIIb	9 (3.2%)
	IVa	2 (0.7%)
	IVb	2 (0.7%)
	V	1 (0.35%)

ARF, acute renal failure; UTI, urinary tract infection

Surgical timing multivariate analyses identified several factors associated with increased operative times. In the docking time model, the use of 5–6 trocars was associated with a significant increase of 1.9 min compared to 3–4 trocars (*p* = 0.009). Regarding console time, the total number of resected lymph nodes was significantly associated with longer duration, with an increase of 1.3 min per node (*p* = 0.007). There were also trends (not statistically significant) for increased console time in patients with Clavien-Dindo ≥ II complications (+ 20 min, *p* = 0.093) and those who received neoadjuvant therapy (+ 32 min, *p* = 0.022). No factors were identified with conversion to laparoscopic or open surgery.

Most dissections were classified as D2 (89.8%). Patients with tumors located in the right colon (cecum, ascending colon, hepatic flexure) had a significantly higher number of resected lymph nodes, with a median of 21 versus 15 in other locations (*p* = 0.006). According to the Gamma regression model, the adjusted mean number of resected nodes was 4.4 higher in right-sided tumors (β = 4.4, 95% CI = 1.2–7.8, *p* = 0.009). The total number of resected nodes was independently associated with the number of positive nodes (β = 0.05, *p* < 0.001). Moreover, patients undergoing D3 or ETM lymphadenectomy had significantly more positive nodes than those treated with CME (+ 2.4 and + 1.8 nodes, respectively; *p* < 0.05).

### Postoperative outcomes

The median hospital stay was 5.5 days (IQR 4–7).

According to the Clavien-Dindo classification, 55 patients (19.2%) had any kind of postoperative complications, mostly minor complications (grade I–II), while 23 patients (8%) presented major complications (grade ≥ III). Anastomotic leakage occurred in 16 patients (5.6%), with 9 with minor AL diagnosed by CT scan and treated with percutaneous drainage or antibiotics. All anastomotic leakages were diagnosed within 30 postoperative days.

A total of 13 patients (4.5%) required surgical reintervention. Of these, 9 (3,1%) were due to major anastomotic leakage or suspected dehiscence. The remaining cases included one pelvic hematoma, one intestinal resection because of bowel lesion, dehiscence of the rectal stump and one evisceration through two trocar sites in a patient with Parkinson’s disease. Readmission within 30 days occurred in 6.7% (19 patients). The perioperative mortality was 0.35% (1 patient), and the 90-day all-cause mortality was 0.7% (2 patients) (Table 4).

**Table 4:** Postoperative complications according to the type of surgical procedure

Type of surgery												
		Abdominoperineal amputation	Subtotal colectomy	Total colectomy +/- reservoir	Right colectomy	Extended right colectomy	Left colectomy	Ventral rectopexy	Anterior resection of the rectum	Ileocecal resection	Segmental resection	Sigmoidectomy
Postoperative Complications	Stoma complications	0	0	0	0	0	0	0	1 (0.4%)	0	0	0
	Evisceration	0	0	0	1 (0.4%)	0	0	0	0	0	0	0
	Paralytic ileus	0	0	0	2 (0.7%)	0	0	0	3 (1.1%)	0	0	0
	Surgical- site infection	0	0	0	0	0	0	0	1 (0.4%)	1 (0.4%)	0	1 (0.4%)
	Anastomotic leak	0	0	1 (0.4%)	5 (1.8%)	0	0	0	8 (2.8%)	1 (0.4%)	0	1 (0.4%)
	Catheter- related bacteraemia	0	0	0	1 (0.4%)	1 (0.4%)	0	0	0	0	0	0
	ARF	0	0	0	2 (0.7%)	0	0	0	0	0	1 (0.4%)	0
	UTI	0	0	1 (0.4%)	3 (1.1%)	0	1 (0.4%)	1 (0.4%)	1 (0.4%)	0	0	1 (0.4%)
	Pneumonia	0	0	0	2 (0.7%)	0	0	0	2 (0.7%)	0	0	0
	Other medical complications	1 (0.4%)	0	0	12 (4.2%)	1 (0.4%)	3 (1.1%)	0	7 (2.5%)	0	0	4 (1.4%)
	No complications	2 (0.7%)	1 (0.4%)	1 (0.4%)	71 (24.9%)	5 (1.8%)	6 (2.1%)	15 (5.3%)	45 (15.8%)	4 (1.4%)	1 (0.4%)	61 (21.4%)

Data are presented as number of patients (%)

ARF: acute renal failure; UTI: urinary tract infection

Multivariate analysis identified several patient-related factors associated with increased postoperative morbidity (Table 5). Patients receiving anticoagulation had a significantly higher risk of developing complications graded Clavien-Dindo ≥ III (OR = 2.73; *p* = 0.047), while ASA ≥ III and male sex showed borderline significance. No robotic setup, configuration, or trocar number was significantly associated with complication severity. Regarding anastomotic leakage, neoadjuvant therapy (OR = 18.3; *p* = 0.005) and anticoagulation (OR = 12.6; *p* = 0.020) were both significantly associated with higher risk of leakage (Table 6).

**Table 5:** Logistic regression results for morbidity

Characteristic	Distribution *n* (%)		Logistic regression		
	<=II *N* = 262	> II *N* = 23	OR	95% CI	*p*-value
*Anticoagulation*					
No	236 (91.2%)	17 (75%)	−	−	
Yes	26 (9.8%)	6 (25%)	2.73	1.01, 7.41	0.047
*ASA Level*					
<=II	191 (73%)	12 (51%)	−	−	
>=III	71 (27%)	11 (49%)	2.48	0.97, 6.34	0.056
*Sex*					
Female	134 (51%)	6 (25%)	−	−	
Male	128 (49%)	17 (75%)	2.26	0.96, 5.60	0.067
*Number of trocars used*					
3–4	66 (25%)	7 (29%)	−	−	
5–6	196 (75%)	16 (71%)	0.83	0.30, 2.45	0.725
*Setup setting*					
2-1-1 (cart between the legs)	60 (23%)	3 (13%)	−	−	
2–2	60 (23%)	4 (16%)	1.27	0.32, 5.17	0.730
3−1	142 (54%)	16 (71%)	0.93	0.21, 4.39	0.928

CI = Confidence Interval, OR = Odds Ratio

**Table 6:** Logistic regression results for anastomotic leak

Characteristic	Distribution *n* (%)		Logistic regression		
	No *N* = 268	YES *N* = 17	OR	95% CI	*p*-value
Arterial hypertension					
No	118 (44%)	5 (29%)	−	−	
Yes	150 (56%)	12 (71%)	5.30	0.72, 65.2	0.131
Neoadyuvant therapy					
No	244 (91%)	10 (57%)	−	−	
Yes	24 (9.0%)	7 (43%)	18.3	2.47, 180	0.005
Anticoagulation					
No	217 (81%)	10 (57%)	−	−	
Yes	51 (19%)	7 (43%)	12.6	1.55, 134	0.020

CI = Confidence Interval, OR = Odds Ratio

## Discussion

This multicenter series, 285 elective colorectal procedures across six Iberian centers, constitutes, to our knowledge, the largest real-world cohort reported with the Hugo™ RAS platform in colorectal surgery. The study demonstrates a low morbidity rate and low operative time, values consistent with previously reported single-center experiences. The scale and heterogeneity of the participating institutions and teams support the reproducibility of outcomes beyond early-adopter centers, extending prior feasibility reports [14–25].

Our findings showed a low conversion (1.1%), major complications (Clavien-Dindo ≥ III) in 8% of patients, and anastomotic leak rate of 5.6%, of which only half required reintervention. Early Hugo™ series in colorectal surgery have shown comparable results. Pietro et al. reported the first colectomies with Hugo™ with no conversions [14] and similarly, Caputo et al. described the first rectal resections with Hugo™ and highlighted the feasibility of pelvic docking strategies without intraoperative complications [17]. Rottoli et al. documented low anterior resections and zero conversions using a multidocking approach [18]. Our outcomes are in line with these early reports and with reference series with other robotic platforms [27], but extend their findings by including a larger, multicenter, and unselected real-world cohort with standardized data collection across six hospitals. These findings suggest that, despite differences in platform architecture, Hugo™ achieves similar safety and quality outcomes once implemented under standardized protocols.

Several technical characteristics of the Hugo™ platform likely contributed to these results. The modular arm-cart design allows tailored trocar geometry and flexible positioning, advantageous for deep pelvic and multiquadrant resections. Published docking-angle frameworks and multi-docking strategies specific to Hugo™ minimize external collisions and optimize access in complex resections [17, 22]. The open console improves communication and teaching during surgery, a key ergonomic advantage repeatedly emphasized in comparative studies versus closed-console systems [2]. These advantages are particularly relevant for technically demanding procedures such as low anterior resections or extended colectomies. In fact, in right-sided resections a higher lymph node yield (median 21 vs. 15; *p* = 0.006), likely reflecting more extensive dissections enabled by the robotic platform.

Operative time (median 210 min) and console time (median 121 min) were consistent with expected durations for robotic colorectal surgery, particularly within a real-world multicenter cohort. Pietro et al. reported the first colectomies with Hugo™, with a mean operative time of 205 min and Rottoli et al. documented median console times of 180 min in low anterior resections and zero conversions using a multidocking approach. Prior series reported console time for rectal resections ranged from 160 to 200 min [14, 17, 18, 21] confirming that the Hugo™ system performs consistent with published robotic colorectal series and surgical timing was influenced primarily by case complexity. Console time increased proportionally with the number of resected lymph nodes and tended to be longer in patients who received neoadjuvant therapy or developed postoperative complications—both of which are markers of more complex surgical scenarios. Docking time was also affected by technical variables, such as the number of trocars used, with procedures involving 5–6 trocars requiring 1.9 min more on average. This finding is consistent with previous modular-system reports and tends to diminish after team familiarization [1, 2, 17, 22]. These findings reflect how procedural complexity, rather than patient factors or platform-related elements, determines overall surgical duration.

The interpretation of anastomotic leakage rates warrants caution given the case mix of the present cohort, with a predominance of colonic resections. As shown in Table 4, anastomotic leakage occurred more frequently after rectal resections than after most colonic procedures, consistent with the higher technical complexity of rectal anastomoses. The rectal leakage rate observed in this series falls within the range reported in contemporary robotic rectal surgery literature, and direct comparisons across studies should therefore be contextualized according to anatomical site, surgical complexity, and program maturity [28]. In terms of morbidity predictors, anticoagulation therapy was the strongest risk factor for both overall severe complications and anastomotic leakage (OR 12.6; *p* = 0.020). Neoadjuvant therapy was also significantly associated with increased leak risk (OR 18.3; *p* = 0.005). In contrast, robotic-related variables—such as configuration, number of trocars, or docking setup—were not associated with complication severity, conversion, or leakage. This pattern mirrors prior Hugo™ reports in general and colorectal surgery, where complication rates ranged from 10% to 20% and were attributed primarily to patient comorbidities and case complexity rather than the system itself [14, 16, 20, 25, 26]. This strongly supports the notion that the robotic platform itself does not introduce additional risk, and that patient-related factors remain the primary drivers of adverse outcomes. The absence of advanced energy devices or robotic staplers did not represent a limitation for achieving favorable outcomes in this series. However, their incorporation into the Hugo™ RAS platform in the near future is expected to further facilitate surgical procedures and potentially optimize operative efficiency and clinical results. The results of the scoping review by Romero-Marcos et al. further contextualize these findings. In their synthesis of over 350 Hugo™ RAS procedures across surgical specialties, the authors confirmed feasibility, reproducibility, and safety, with conversion rates below 2% and major complication rates under 10% [26]. Our multicenter data reinforce these conclusions, providing quantitative validation within the colorectal domain and across a broader real-world setting.

From an implementation perspective, the present study demonstrates that structured training and standardized setup enable efficient adoption, even in teams initially naïve to robotics. Previous work by Romero-Marcos et al. showed that non-robotic colorectal teams achieved proficiency within their first 10–15 Hugo™ cases after simulator and mentored training [16]. Our multicenter experience mirrors this trajectory, with consistent outcomes across centers and a low conversion rate despite different levels of prior robotic experience.

Strengths of this study include its multicenter design with real-world data, standardized definitions for complications and outcomes, and a high level of data completeness. Nevertheless, several limitations should be acknowledged. The retrospective design and the absence of a control group (e.g., laparoscopic or other robotic platforms) limit causal inference and direct benchmarking. In addition, all participating surgeons were robot-naïve at the time of Hugo™ RAS implementation, and procedures were performed during the early adoption phase of each center’s program; therefore, outcomes may reflect a mixture of initial learning-curve and more consolidated experience. Despite these limitations, the low conversion rate and acceptable perioperative outcomes observed across centers suggest that safe and reproducible implementation of the Hugo™ RAS platform is achievable from the early phases of adoption when structured training and standardized protocols are applied. As noted in systematic reviews, early Hugo™ studies may be subject to reporting bias favoring positive experiences [29]. Future research should include prospective comparative trials, long-term oncologic outcomes, and cost-effectiveness analyses to further define the clinical and economic role of this platform.

In summary, the COLOROBOT IBERICA study provides robust multicenter evidence confirming that the Hugo™ RAS platform is safe, effective, and reproducible for colorectal surgery in real-world practice. Its modular hardware, open-console ergonomics, and flexible docking configurations allow broad integration across different institutions and surgeon profiles. When combined with structured training and standardized protocols, the platform achieves consistent perioperative outcomes, supporting the feasibility and safe implementation of Hugo™ RAS in routine colorectal practice.

## Data Availability

The datasets generated and analyzed during the current study are available from the corresponding author upon reasonable request.
